# Comment on: A novel method to assess airway eosinophilia using sputum plugs

**DOI:** 10.1183/23120541.00428-2026

**Published:** 2026-10-26

**Authors:** Einat Fireman-Klein, Elizabeth Fireman

**Affiliations:** 1 Pulmonary Division, Carmel Medical Center, Haifa, Israel; 2 Technion-Israel Institute of Technology, The Ruth and Bruce Rappaport Faculty of Medicine, Haifa, Israel; 3 The Department of Environmental and Occupational Health at the School of Public Health, Gray Faculty of Medical and Health Sciences, Tel Aviv University, Tel-Aviv, Israel

## Abstract

We read with great interest the article by Patel *et al.* [1] entitled “A novel method to assess airway eosinophilia using sputum plugs” published *ERJ Open Research*.

## Abstract 2

**A simplified method to detect eosinophils from induced sputum** https://bit.ly/4eD4Ik2

*To the Editor:*

We read with great interest the article by Patel *et al.* [1] entitled “A novel method to assess airway eosinophilia using sputum plugs” published *ERJ Open Research*.

The paper highlights a practical limitation of the induced sputum technique. Although this method represents a safe and noninvasive approach for assessing eosinophilic airway inflammation in asthma, its routine use remains limited due to the labour-intensive sample processing required, which necessitates trained personnel and specialised laboratory facilities.

In their study, the authors proposed an alternative approach in which sputum plugs were fixed in 10% formalin and embedded in paraffin, termed the SSIMPLE (Sputum-Minimising Processing, Maximising Clinical Outcomes) method. The resulting formalin-fixed, paraffin-embedded (FFPE) sputum plugs were sectioned, stained with haematoxylin and eosin, and independently evaluated by four blinded observers, who performed differential cell counts on 400 nonsquamous cells.

As is described in the methods section, the alternative method should be processed in a pathological laboratory well equipped by fixation facilities (30×50-mm biopsy mesh bags (VWR #10809-874), secured in tissue cassettes (Leica Biosystems #3802646), a microtome (Leica RM2255), haematoxylin and eosin according to the standard operating procedure of the Hamilton Regional Laboratory Medicine Program.

Processing sputum plugs as FFPE material with haematoxylin–eosin staining represents a histological rather than cytological approach, and may influence cell morphology, staining properties and differential cell counts, thereby limiting direct comparison with the established body of induced sputum literature, which is based on cytological processing of selected sputum plugs and May–Grünwald–Giemsa-stained cytospin preparations according to European Respiratory Society/American Thoracic Society (ERS/ATS) methodology [2]. Furthermore, formalin fixation and histological processing are known to alter protein structure, antigenicity and staining characteristics, which may affect cellular identification compared with conventional cytological preparations [3].

In contrast, we previously published the paper “Simplified detection of eosinophils in induced sputum” [4] in which we demonstrated step-by-step simplification of the sputum plug-selection method recommended by the ERS/ATS Task Force, considered the “gold standard”, originally described by Popov *et al*. [5].

To simplify sputum processing and avoid the use of equipment not routinely available in standard laboratories, selected sputum plugs were treated with diluted Sputolysin and incubated at room temperature for 30 min instead of 15 min at 37°C. The reaction was neutralised to prevent further lysis and the sample was again sedimentated at room temperature for 30 min. During both incubation steps, the sample should be agitated vigorously and the incubation time may be prolonged if required. After removal of the supernatant, the resulting pellet allowed preparation of a haematological smear stained with Giemsa, enabling eosinophil count as recommended by the ERS/ATS Task Force (figure 1).

**FIGURE 1:**
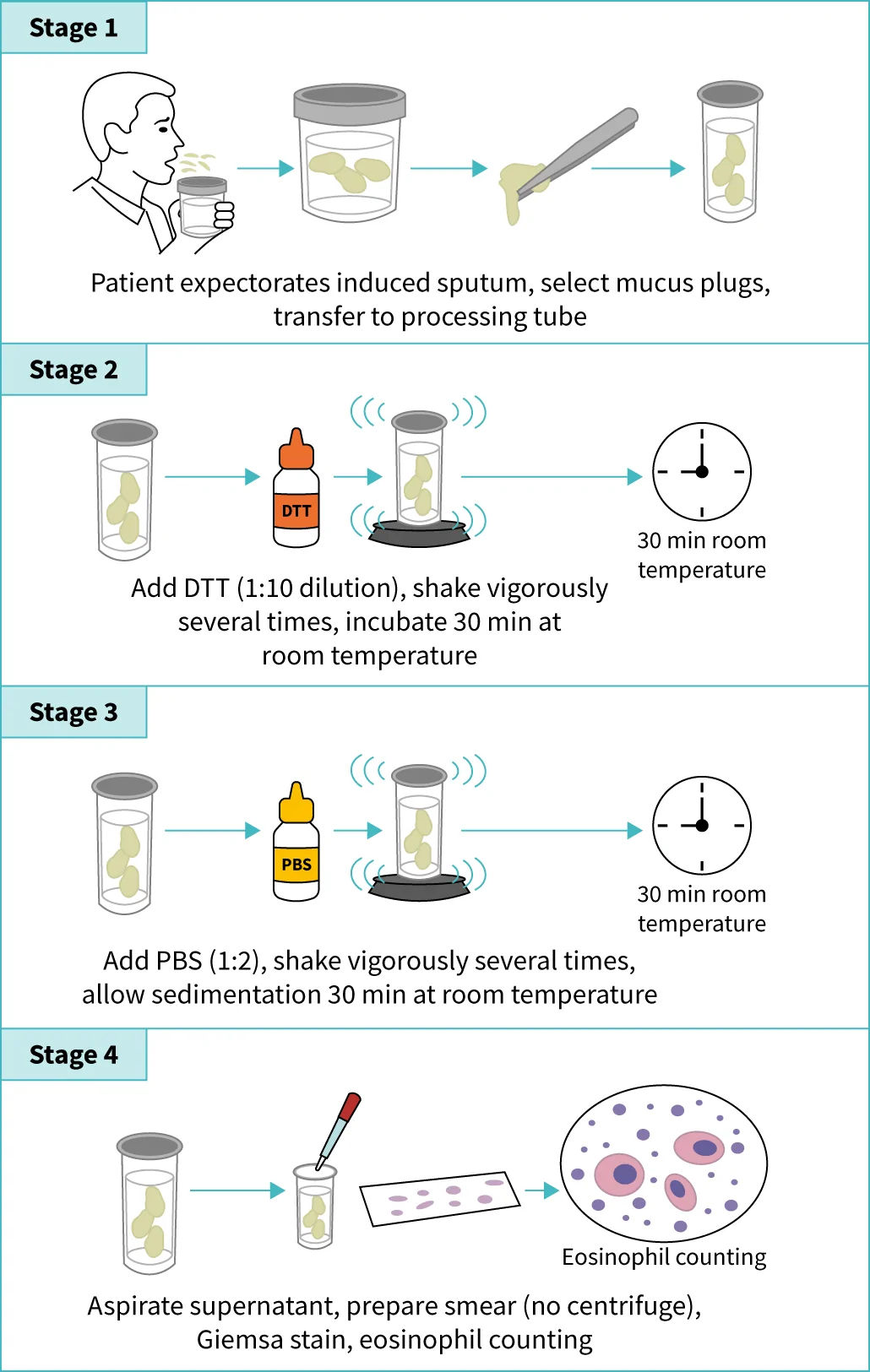
A simplified method for the detection of sputum eosinophilia. DTT: dithiothreitol.

In the era of biologic therapies, prediction of clinical remission in patients with severe asthma cannot rely solely on indirect surrogate biomarkers of type-2 inflammation, such as blood eosinophil counts [6]. Furthermore, a relatively clinically weak correlation was shown between airway eosinophilia and peripheral blood eosinophil counts for COPD treated with biologics [7].

This emphasises the need to improve access to a simplified method for induced sputum testing. We believe that this simplified method may facilitate the use of sputum analysis in routine practice and thereby support a more personalized approach to biologic treatment.
